# Galectin-3 as a marker of interstitial atrial remodelling involved in atrial fibrillation

**DOI:** 10.1038/srep40378

**Published:** 2017-01-12

**Authors:** Diana Hernández-Romero, Juan Antonio Vílchez, Álvaro Lahoz, Ana I. Romero-Aniorte, Eva Jover, Arcadio García-Alberola, Rubén Jara-Rubio, Carlos M. Martínez, Mariano Valdés, Francisco Marín

**Affiliations:** 1 Department of Cardiology, IMIB-Arrixaca from Murcia, Spain; 2 Department of Clinical Analysis, IMIB-Arrixaca from Murcia, Spain; 3 Intensive Care Unit, IMIB-Arrixaca from Murcia, Spain; 4 CIBERehd, Instituto de Salud Carlos III, Madrid, Spain

## Abstract

Remodelling in the atria could appear as a result of hypertension, diabetes or ischaemic heart disease. Galectin-3 (Gal-3) is a mediator of profibrotic pathways and a potential biomarker of cardiac remodelling. We prospectively recruited consecutive patients undergoing elective cardiac surgery. Preoperative Gal-3 levels were determined from serum samples, and the presence of fibrosis was assessed from atrial appendage tissue samples obtained during cardiac surgery. We included 100 patients with aortic valve or ischaemic heart diseases and 15 controls with permanent AF. Gal-3 levels were associated with sex, left atrial volume, previous cardiac disease, diabetes mellitus, hypertension, NYHA and NT-proBNP. We observed differences in serum Gal-3 concentrations between patients and controls with permanent AF (p = 0.020). We performed ROC curves related to fibrosis and established a cutoff point for Gal-3 >13.65 ng/ml. Multivariate analyses showed previous cardiac disease, NYHA scale and high Gal-3 to be independent predictors of fibrosis. After adjustment for confounding factors, atrial fibrosis remained the only independent factor for the development of AF (p = 0.022). High Gal-3 serum levels predict fibrosis of the atrial appendage. NYHA scale and previous cardiac disease were also associated with tissue fibrosis in patients undergoing surgery. Atrial fibrosis was the only independent predictor for post-operative AF occurrence in our model after correcting for confounding factors.

Changes in atrial function and structure are known as atrial remodelling, which contributes to the development of atrial fibrillation (AF)^1^. AF is associated with an increased morbidity and mortality after cardiac surgery and a longer hospital length of stay^2^,^3^. A significant association between fibrosis, inflammation, oxidative stress and the development, recurrence and perpetuation of AF has been hypothesized^4^.

AF after cardiac surgery occurs in approximately 20–50% of patients undergoing cardiac surgery^5^. Numerous predisposing factors such as advanced age, hypertension, diabetes, left atrial enlargement, left ventricular hypertrophy, or intra-operative and post-operative factors such as atrial injury or ischaemia, have been associated with the development of post-operative AF^6^.

Galectin-3 (Gal-3) is a beta-galactoside binding lectin that appears to be a mediator of cardiac fibrosis in a number of recent experimental studies. Gal-3 has been reported as a mediator of profibrotic pathways and as a potential biomarker of adverse cardiac remodelling^7^. Gal-3 is expressed by activated macrophages and induces cardiac fibroblasts to proliferate and deposit type I collagen in the myocardium^8^,^9^. This protein is linked to areas of fibrosis, suggesting an active role in modulation of the extracellular matrix. Therefore, Gal-3 appears to link pathways of inflammation and fibrosis, and it may contribute to the development of heart failure. Its role in atrial remodelling has scarcely been studied, and it was the main purpose for the present manuscript.

## Results

We included 100 patients with predominantly aortic valve (n = 42) or ischaemic heart (n = 58) diseases and 15 controls with permanent AF, all of whom underwent cardiac surgery. Twenty-nine patients (29%) developed post-surgical AF, of whom 13 occurred during the Intensive Care Unit stay. Aortic patients showed a higher rate of AF development than coronary patients (41.5% vs 20.7%, p = 0.026). Patients developing AF had a longer stay in the Intensive Care Unit (p = 0.008) as well as longer overall hospitalization stays (p = 0.005).

We observed differences in serum Gal-3 concentrations between patients and controls with permanent AF (14.25 ± 4.15 vs 17.61 ± 6.84 ng/mL; p = 0.020) (Table 1). The maximum and minimum Gal-3 levels were 23.10–7.00 for patients and 24.30–8.00 for controls. No differences between aortic and coronary patients (14.71 ± 4.34 vs 13.91 ± 4.02 ng/mL; p = 0.402) were observed. In a univariate regression model, Gal-3 was significantly associated with age, sex, left atrial volume, previous cardiac disease, *diabetes mellitus*, hypertension, and NYHA functional class. In multivariate analysis, only sex, previous cardiac disease and *diabetes mellitus* remained independent predictors for Gal-3 values (all p < 0.05; Table 2). We evaluated correlations with NT-proBNP values, an established biomarker of wall stress and cardiac remodelling, and we found a significant positive correlation: r = 0.226, p = 0.045.

### Gal-3 levels as a predictor for atrial fibrosis

**Figure 1:**
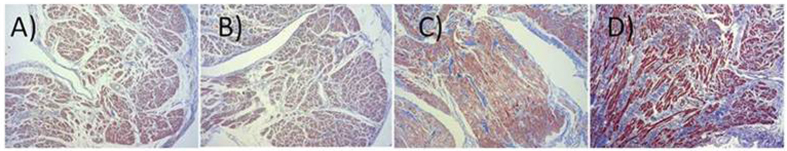
Classification of the myocardial fibrosis assessed by Masson’s trichrome staining infiltration grades: grade 0 (**A**), 1 (**B**), 2 (**C**) and 3 (**D**).

**Figure 2:**
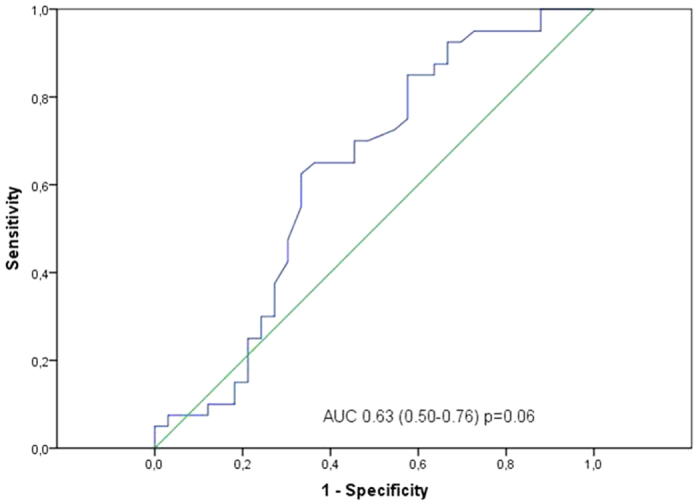
Receiver Operating Characteristic (ROC) for evaluation of GAL-3 levels related to AF development in patients undergoing cardiac surgery.

When evaluating atrial appendage interstitial fibrosis using Masson’s trichrome as indicated, 5 (4.5%) tissue samples showed low fibrosis (connective tissue infiltration Grade 1), 37 (40.2%) showed medium fibrosis (connective tissue infiltration Grade 2), and 50 (54.3%) showed intensive interstitial fibrosis (connective tissue infiltration Grade 3) (Fig. 1A–D). Eight samples were not evaluable due to different technical reasons. We performed ROC curves related to high-grade fibrosis (AUC: 0.630 ± 0.069 (CI95%: 0.494–0.762); p: 0.06; Fig. 2) and established a cutoff point for Gal-3 >13.65 ng/ml. Univariate analysis showed that age, previous cardiac disease, septal thickness, NYHA scale, creatinine clearance (CrCl) and high Gal-3 were associated with fibrosis (Table 3). Likewise, in multivariate analyses, we observed that previous cardiac disease, NYHA scale and high Gal-3 [OR (95%CI): 4.37 (1.16–16.41), p = 0.029; 2.93 (1.26–6.85), p = 0.013 and 3.29 (1.07–10.11), p = 0.037, respectively] remained independent predictors of fibrosis (Table 3).

### Interstitial fibrosis in the prediction of atrial fibrillation occurrence

We also proposed to evaluate the incidence of tissue remodelling in the occurrence of AF in patients after cardiac surgery. Demographic factors including age and sex, clinical variables such as body mass index, left atrial volume, EuroSCORE index or type of surgery, and interstitial atrial fibrosis were associated with AF occurrence in a logistic regression analysis [OR (95%CI): 1.09 (1.00–1.11), p = 0.048; 4.96 (1.84–13.36), p = 0.031; 1.09 (0.98–1.21), p = 0.123; 1.06 (1.00–1.11), p = 0.042, 1.26 (1.03–1.54), p = 0.025; 2.61 (1.08–6.32), p = 0.034 and 2.18 (0.88–5.44), p = 0.094, respectively]. After adjustment for potential confounding factors, only atrial remodelling evaluated as tissue atrial fibrosis remained an independent factor for AF development [OR (95%CI): 3.77 (1.20–11.76), p = 0.022; Table 4] in our multivariate model. When reanalyzing by excluding subsequent confounding factors to avoid a problem of “over-fitting,” only the atrial fibrosis remained as an independent factor (data not shown).

## Discussion

Gal-3 has been reported to be a biomarker for cardiac fibrosis. Our results support previous studies since we found a clear association between Gal-3 serum levels and interstitial fibrosis measured in the right atrial appendage obtained by resection.

Fibrosis is a result of structural remodelling, and it has been proposed as the main arrhythmogenic substrate perpetuating AF^10^. In our cohort, 20% of patients showed clinical AF during their hospitalization stay after coronary surgery. This finding is consistent with previous data indicating AF occurrence in approximately 20–50% of patients undergoing cardiac surgery (5). Pre-surgical levels of Gal-3 suggest an active remodelling process in the atrial appendage tissue prior to the surgical intervention. In addition, we also found a positive correlation between Gal-3 and NT-proBNP levels as a biomarker of established wall stress and cardiac remodelling, supporting our hypothesis. In previous studies, we observed that pre-surgical hsTnT^11^ and vWF levels^12^ were indicators of ongoing subclinical myocyte damage, endothelial dysfunction and remodelling in the atria. Both biomarkers resulted were therefore associated with AF development in patients undergoing cardiac surgery. Here, we found an association between Gal-3 levels and fibrosis when analyzing all the available samples. Our results indicate that Gal-3 pro-fibrotic effects and interstitial atrial remodelling are converging processes in fibrosis development. Whether or not this fibrosis is the responsible cause for AF development, is an assumption that we cannot demonstrate with our results, but is supported by the clear association between fibrosis and AF development. Our data describing higher values of left atrial volume and septal thickness in both patients presenting with intensive fibrosis and in patients who developed AF also reinforce the same idea. In this context, surgery would act as a trigger for the development of AF in a predisposed environment involving highly remodelled tissue.

In addition, we found higher Gal-3 levels in a positive control cohort of permanent AF patients, supporting our hypothesis. Recent studies have found a correlation between Gal-3 levels and atrial remodelling, including the extent of left atrial fibrosis and atrial electromechanical properties^13^.

This study is limited by its observational design; we could explore only associations, and no causality is implied. The recruitment protocol did not guarantee the exclusion of patients with previously silent AF from the study. Although Gal-3 level has been proposed as a biomarker of fibrosis in cardiovascular diseases, we cannot ignore possible changes in Gal-3 levels over time. Another limitation is related to the studied tissue samples, as we had no access to left atrial appendage tissue.

In conclusion, we can summarize that Gal-3 levels are higher in controls with permanent AF versus patients without previous known AF undergoing cardiac surgery. High Gal-3 serum values predict fibrosis of the right atrial appendage. Other clinical factors such as NYHA scale and previous cardiac disease were also associated with the presence of fibrosis in patients undergoing surgery. Atrial fibrosis was the only independent predictor for post-operative AF occurrence in our model, even after correcting for confounding factors.

## Methods

### Patients

We prospectively recruited consecutive patients undergoing elective cardiac surgery with cardiopulmonary bypass from November 2010 until February 2012. We excluded patients with previous AF (paroxysmal or permanent), unstable angina, hepatic or renal failure (creatinine clearance <50 ml/min), and chronic inflammatory or neoplasic diseases. Patients undergoing urgent surgery and those with a previous history of pacemakers, infectious endocarditis and those undergoing AF-related surgery were also excluded.

We documented AF during the post-operative period in the Intensive Care Unit by continuous 3-derivations telemetry and by a Holter device once the patient was in the hospitalization cardiac surgery unit. Holter monitoring was extended until a maximum of 10 days after surgery. In addition, a 12 derivations electrocardiogram was performed in symptomatic patients and daily during the hospitalization. AF development was defined as an episode of AF lasting for more than 2 minutes in any of the ECG registries.

All echocardiographic measurements were performed off-line by the same accredited cardiologist who was unaware of clinical and laboratory data^14^. Left atrial volume was calculated according to the ellipsoid model that assumes that the left atrium can be adequately represented as a prolate ellipsoid with a volume of 4p⁄3(L⁄2)(D1⁄2)(D2⁄2), where L is the long-axis (ellipsoid) and D1 and D2 are the orthogonal short-axis dimensions^15^. Left atrial volume calculations were indexed to body surface area calculated according to Gehan and George^16^.

The study was carried out according to the principles of the Declaration of Helsinki and was approved by the Ethics Committee of the Hospital Universitario Virgen de la Arrixaca. All the included patients gave informed consent to participation.

### Blood samples and laboratory assays

Venepuncture was performed the morning of cardiac surgery with the patient fasting for >12 hours. We collected samples immediately before cardiac surgery. Plasma fractions were obtained by centrifugation for 15 minutes at 3500 × *g*. Aliquots were stored at −40 °C to allow batch analysis in a blinded fashion.

Preoperative Gal-3 levels were determined in defrosted serum samples by ELFA (Enzyme-Linked Fluorescent Assay) in a MiniVidas analyzer (BioMérieux^®^, France). The inter-assay and intra-assay coefficients of variation were 6.5% and 1.6%, respectively. The measured range was 3.3–100 ng/mL, the lower limit of detection was 2.2 ng/mL and the limit of quantification was 3.3 ng/mL. Levels of preoperative NT-proBNP were measured as described elsewhere^11^.

### Obtaining and staining right atrial appendage tissue

Atrial appendage tissue was obtained during surgery by cannulation for the extracorporeal circulation. This cannulation was performed directly into the right atria with non-absorbable suture in a “tobacco bag” shape. To provide adequate cannula apposition, the bag was opened and cut. The remaining appendage tissue was collected for the tissue study objectives.

All recruited subjects gave their informed consent to participate in the study. All surgical procedures were performed under cardiopulmonary bypass, with mild hypothermia (30 °C), cardioplegic arrest of the heart and left ventricular (LV) venting through the right superior pulmonary vein. We used anterograde and retrograde cold intermittent blood cardioplegia (Cardi-Braun^®^; B-Braun, Inc., Barcelona, Spain) for myocardial protection.

The tissue samples were processed, paraffin embedded and cut into 2–3 μm sections. For histochemical evaluation of connective tissue infiltration within the myocardial tissues, a Masson’s trichrome staining was performed on sections from affected specimens by an automatized staining system (Dako Artisan, Dako, Carpinteria, California, USA) following the manufacturer’s recommendations. The degree of connective tissue infiltration was measured using a qualitative scale from 0 to 3 (0 was negative; 1, mild; 2, medium; and 3, high infiltration) at the location within the tissue (perivascular or interstitial fibrosis). All assessments were blinded and performed twice to ensure the repeatability of the results. The analysis was made using an Axio Scope A1 transmitted-light microscope (Carl Zeiss, Jena, Germany).

### Statistical analysis

Categorical variables are presented as counts (percentages), while continuous variables are presented as the mean ± SD (standard deviation) or median (25th–75th percentiles), as appropriate. The Kolmogorov-Smirnov test was used to check for normal distribution of continuous data.

Variables associated with Gal-3 values were studied by linear regression analysis (stepwise mode). We constructed areas under the receiver-operator characteristic (ROC) curve for Gal-3 related to high grade fibrosis. The cutoff point with the best sensitivity and specificity was chosen for each case, as assessed by ROC curves. Gal-3 levels were dichotomized as ‘low’ or ‘high’ according to whether the circulating levels were under or over the calculated threshold (cutoff). This dichotomy for Gal-3 levels was assessed via the logistic regression model to explore the overall association between fibrosis in tissues obtained by myectomy and Gal-3 values. We considered fibrosis to be intensive when the degree of connective tissue infiltration was 2 or 3 with Masson’s trichrome stain. Logistic regression analyses were performed to assess the predictive variables for AF development including fibrosis. All p values < 0.05 were accepted as statistically significant. Statistical analysis was performed using SPSS 19.0 for Windows (SPSS, Inc., Chicago, IL, USA).

## Additional Information

**How to cite this article**: Hernández-Romero, D. *et al*. Galectin 3 as marker of interstitial atrial remodeling involved in atrial fibrillation. *Sci. Rep.* **7**, 40378; doi: 10.1038/srep40378 (2017).

**Publisher's note:** Springer Nature remains neutral with regard to jurisdictional claims in published maps and institutional affiliations.

## Figures and Tables

**Table 1:** Baseline characteristics of included patients (n: 100).

Clinical or demographic variable	%, mean ± sd or median (IQR)
Age (years)	65.1 ± 9.5
Male	77
Coronary patient	58
Aortic Valvular patient	42
Smoking habit	23
Hypertension	70
Hypercholesterolemia	65
*Diabetes mellitus*	47
DM insulin-dependent	12
DM under oral treatment	38
Previous pulmonary chronic obstructive disease	8
Previous thyroid disease	6
Previous stroke	9
NYHA	2 (1–2)
EuroSCORE	4 (2–5)
Left atrial diameter (mm)	40.71 ± 5.80
Left atrial volume (Ellipsoidal; mm^3^)	27.08 ± 8.81
Aortic clamp time (min)	46.3 ± 16.8
Cardiopulmonary bypass pump time (min)	90.37 ± 28.76
AF development in the Intensive Care Unit	13
Total hospitalization time (days)	9 (8–16)
Stay in the Intensive Care Unit (days)	2 (2–4)
Stay in the Cardiology Department (days)	7 (5–14)
Galectin concentration (mg/mL)	14.25 ± 4.15

EuroSCORE: The European System for Cardiac Operative Risk Evaluation. NYHA: New York Heart association functional class.

**Table 2:** Linear regression (stepwise mode) analysis for the predictors of Galectin-3 values.

	Univariate analysis B-coefficient (95% CI); p value	Multivariate analysis B-coefficient (95% CI); p value
Age	0.11 (0.02–0.21); 0.023	0.140
Gender, male	2.75 (0.65–4.85); 0.011	2.88 (0.81–4.95); **0.007**
Left atrial volume	0.10 (−0.10–0.21); 0.073	0.127
Previous cardiac disease	−2.56 (−4.67 to −0.45); 0.018	−2.19 (−4.31 to −0.07); **0.044**
*Diabetes mellitus*	2.37 (0.58–4.17); 0.010	2.08 (0.29–3.87); **0.024**
Hypertension	2.66 (0.69–4.63); 0.009	0.050
NYHA scale	1.01 (0.−0.35–2.37); 0.144	0.151
Smoking habit	−0.013 (−2.20–2.18); 0.991	
CrCl	−1.06 (−5.84–3.72); 0.660	
Dyslipidaemia	0.69 (−1.30–2.67); 0.494	

NYHA: New York Heart association functional class CrCl: Creatinine clearance.

**Table 3:** Logistic regression analysis for fibrosis presence.

	Univariate analysis OR (95%CI); p value	Multivariate analysis OR (95%CI); p value
Age	1.03 (0.99–1.08); p = 0.105	1.21 (1.15–3.08); p = 0.523
Gender, male	1.15 (0.04–3.09); p = 0.770	
Hypertension	1.28 (0.52–3.13); p = 0.580	
*Diabetes mellitus*	1.12 (0.53–2.81); p = 0.622	
Previous cardiac disease	2.44 (0.96–6.19); p = 0.059	4.37 (1.16–16.41); **p = 0.029**
Left atrial volume	0.99 (0.95–1.04); p = 0.949	
Septum thickness	16.85 (2.01–136.36); p = 0.008	8.23 (0.30–233.0); p = 0.211
Dyslipidaemia	1.58 (0.66–3.76); p = 0.295	
Smoking habit	1.03 (0.38–2.79); p = 0.947	
NYHA scale	2.05 (1.09–3.83); p = 0.024	2.93 (1.26–6.85); **p = 0.013**
CrCl	0.98 (0.96–1.00); p = 0.062	0.99 (0.97–1.01); p = 0.689
Galectin-3 >13.65 ng/mL	3.33 (1.26–8.76); p = 0.015	3.29 (1.07–10.11); **p = 0.037**

NYHA: New York Heart association functional class CrCl: Creatinine clearance.

**Table 4:** Logistic regression for the prediction of AF occurrence.

	Univariate		Multivariate	
	HR (CI95%)	p	HR (CI95%)	p
Age	1.09 (1.00–1.11)	**0.048**	1.00 (0.93–1.08)	0.951
Male sex	4.96 (1.84–13.36)	**0.031**	2.14 (0.58–7.88)	0.252
Hypertension	1.51 (0.56–4.03)	0.415		
Hypercholesterolemia	2.05 (0.77–5.42)	0.150		
*Diabetes mellitus*	1.31 (0.55–3.10)	0.546		
Body mass index	1.09 (0.98–1.21)	0.123	1.06 (0.92–1.22)	0.443
Indexed left atrial volume	1.06 (100–1.11)	**0.042**	1.03 (0.96–1.10)	0.380
Left ventricular ejection fraction	0.99 (0.96–1.03)	0.797		
Clamping time	0.98 (0.96–1.01)	0.235		
Cardiopulmonary pump time	0.99 (0.98–1.01)	0.679		
Type of surgery (valve surgery vs CABG)	2.61(1.08–6.32)	**0.034**	2.73(0.63–11.86)	0.181
EuroSCORE	1.26 (1.03–1.54)	**0.025**	1.28 (0.91–1.79)	0.150
Interstitial atrial appendage fibrosis	2.18 (0.88–5.44)	0.094	3.77 (1.20–11.76)	**0.022**

CABG: coronary artery bypass grafting; EuroSCORE: The European System for Cardiac Operative Risk Evaluation; ACE: angiotensin-converting enzyme; ARBs: angiotensin receptor blockers; CA: calcium antagonists.
